# Supplementary material for: Isolation and Characterization of Yeast with Benzenemethanethiol Synthesis Ability Isolated from Baijiu Daqu
Source: Foods. 2023 Jun 23;12(13):2464. doi: 10.3390/foods12132464 (PMC10340341; doi:10.3390/foods12132464)
Supplement: Supplementary file 1 [file foods-12-02464-s001.zip › foods-2424974-supplementary.pdf]

Supplementary information for

Isolation and Characterization of Yeast with Benzenemethanethiol Synthesis Ability Isolated from Baijiu Daqu

Guihu Zhang [1,2,3], Peng Xiao [1,2,3], Youqiang Xu [1,2,3], Honghua Li [1,2], Hehe Li [1,2,3,*], Jinyuan Sun [1,2,3], Baoguo Sun [1,2,3]

[1] Key Laboratory of Geriatric Nutrition and Health (Beijing Technology and Business University), Ministry of Education, Beijing 100048, China

[2] China Food Flavor and Nutrition Health Innovation Center, Beijing Technology and Business University, Beijing 100048, China

[3] Key Laboratory of Brewing Molecular Engineering of China Light Industry, Beijing Technology and Business University, Beijing 100048, China

* Correspondence:

H. Li, Beijing Technology & Business University. No. 33, Fucheng Road, Haidian District, Beijing 100048, China. E-mail: xyzhehe@126.com

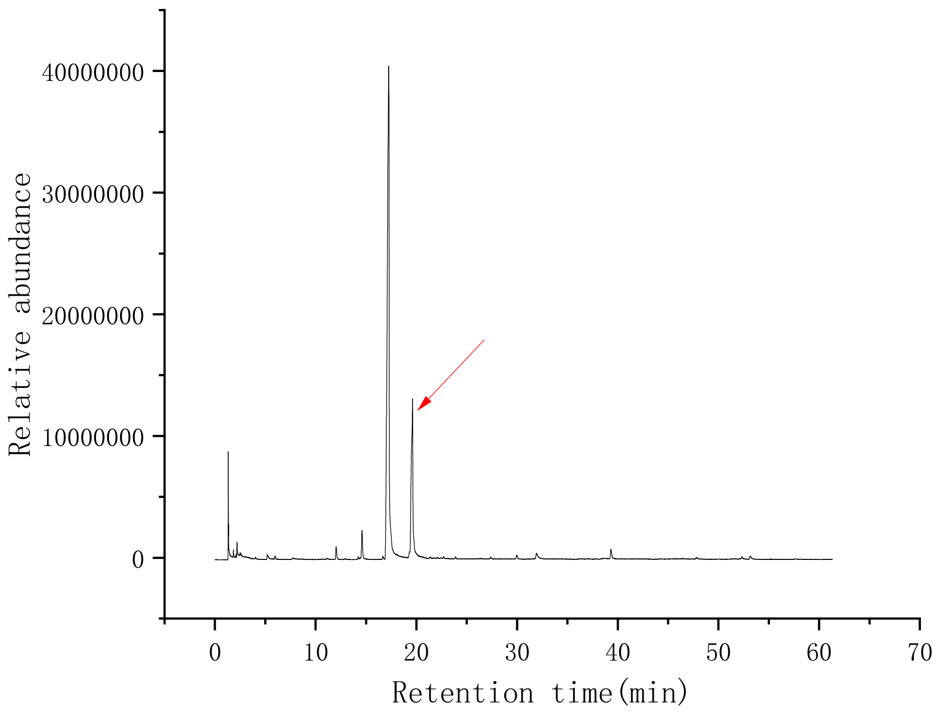

Figure S1. GC-SCD chromatogram of benzenemethanethiol of *Saccharomyces cerevisiae* J14.

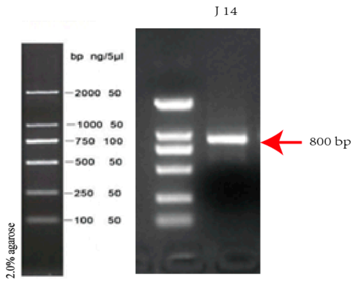

Figure S2. Electrophoresis of 5.8S rDNA of strain J14.

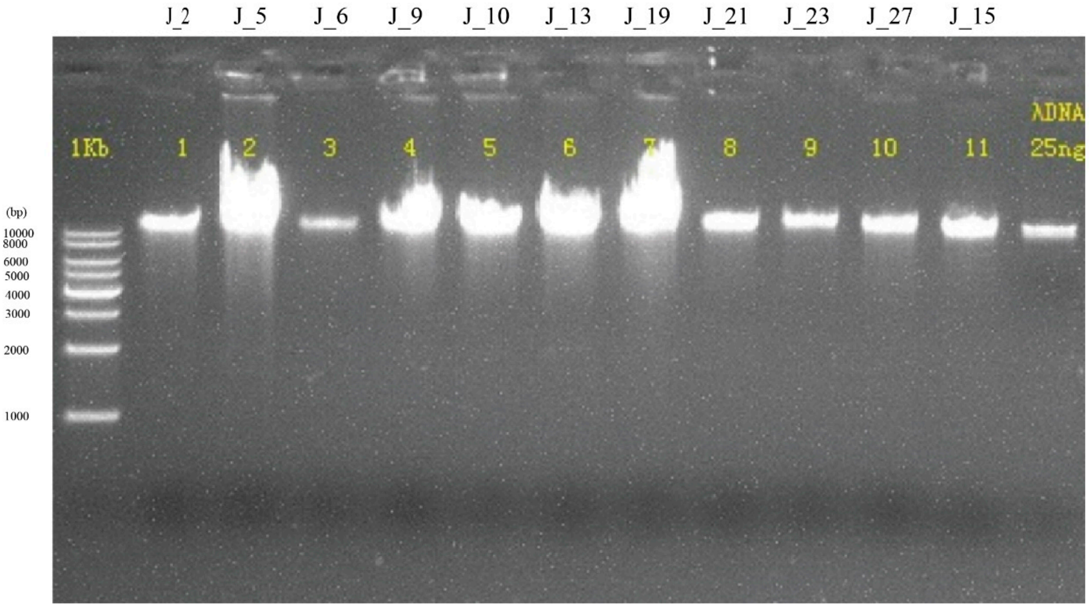

Figure S3. The photo of genome extraction of 11 yeast capable benzenemethanethiol biosynthesis.
